# GETV nsP2 plays a critical role in the interferon antagonism and viral pathogenesis

**DOI:** 10.1186/s12964-023-01392-x

**Published:** 2023-12-18

**Authors:** Chunxiao Mou, Hui Meng, Kaichuang Shi, Yanmei Huang, Meiqi Liu, Zhenhai Chen

**Affiliations:** 1 College of Veterinary Medicine, Yangzhou University, No.12 Wen-hui East Road, Yangzhou, JS225009 Jiangsu Province People’s Republic of China; 2 Joint International Research Laboratory of Agriculture and Agri-Product Safety, the Ministry of Education of China, Yangzhou University, Yangzhou, 225009 Jiangsu Province People’s Republic of China; 3 Jiangsu Co-Innovation Center for Prevention and Control of Important Animal Infectious Diseases and Zoonoses, Yangzhou University, Yangzhou, 225009 Jiangsu Province People’s Republic of China; 4 Guangxi Center for Animal Disease Control and Prevention, Nanning, GX China

**Keywords:** Getah virus, nsP2 protein, Interferon, Nuclear localization, Viral pathogensis

## Abstract

Getah virus (GETV) was becoming more serious and posing a potential threat to animal safety and public health. Currently, there is limited comprehension regarding the pathogenesis and immune evasion mechanisms employed by GETV. Our study reveals that GETV infection exhibits the capacity for interferon antagonism. Specifically, the nonstructural protein nsP2 of GETV plays a crucial role in evading the host immune response. GETV nsP2 effectively inhibits the induction of IFN-β by blocking the phosphorylation and nuclear translocation of IRF3. Additionally, GETV nsP2 hinders the phosphorylation of STAT1 and its nuclear accumulation, leading to significantly impaired JAK-STAT signaling. Furthermore, the amino acids K648 and R649, situated in the C-terminal region of GETV nsP2, play a crucial role in facilitating nuclear localization. Not only do they affect the interference of nsP2 with the innate immune response, but they also exert an influence on the pathogenicity of GETV in mice. In summary, our study reveals novel mechanisms by which GETV evades the immune system, thereby offering a foundation for comprehending the pathogenic nature of GETV.

Video Abstract

**Supplementary Information:**

The online version contains supplementary material available at 10.1186/s12964-023-01392-x.

## Introduction

Getah virus (GETV), one of the *Alphavirus* genus and the *Togaviridae* family member, was first isolated from the Culex gelidus mosquitoes of Malaysia and Japan in the 1950s [1]. Since then, GETV has reportedly outbreak worldwide, such as East Eurasia and South-East Asian countries [2]. GETV could be amplified through the mosquito–vertebrate host–mosquito cycle and infected horses, pigs and cattle [3]. GETV infected swine of all ages, especially, newborn piglets, caused high morbidity, mortality, and serious economic losses [4]. Moreover, serologic surveys and virus isolation showed that the infection might occur in multiple other vertebrate species, including cattle, monkeys, goats, rabbits, kangaroos, poultry, wild birds, and human beings [5].

GETV is an enveloped, single-stranded positive-strand RNA virus [6]. The full-length viral genome is 11–12 kb, which contains two open reading frames (ORFs) that encode a nonstructural and a structural polyprotein, also includes the methylated cap structure of 5’ end and has a poly (A) tails in the 3’ end [7]. The nonstructural polyprotein is cleaved by viral and host factors into 4 nonstructural proteins (nsP1-4), which are responsible for viral RNA transcription and replication. NsP1 is a methyltransferase that is linked to cellular membranes. NsP2 encompasses the viral helicase, protease, and a hypothetical C-terminal methyltransferase domain, and forms associations with numerous host proteins, thereby effectively suppressing host cell protein synthesis. NsP3 is a phosphoprotein that recruits the host factor G3BP, thereby impeding the formation of cellular stress granules [8]. Lastly, nsP4 functions as the viral RNA-dependent RNA polymerase. The second ORF encodes five structural proteins (C, E3, E2, 6 K and E1) [7, 9]. The C protein is employed in the packaging of the viral nucleic acid, while the 6 K protein actively engages in the assembly and budding phases of the viral particles on the surface of infected cells. Additionally, the E1 and E2 proteins facilitate the entry of the virus into host cells. Furthermore, the concerted efforts of the 6 K and E3 proteins are instrumental in transporting the precursor membrane protein to the endoplasmic reticulum (ER) [10].

The role of innate immunity in the response to alphavirus infection is significant [11]. The cytokine IFN-β plays a critical role in the antiviral response and activation of the innate immune system. In mammalian cells, pathogen-associated molecular patterns (PAMPs) of invading viruses are detected by the pattern-recognition receptors (PRRs), such as the membrane-bound receptors (TLR3 and TLR7) and the cytosolic receptors (RIG-I and MDA5) [12, 13]. Through activating the downstream cascades, the transcription factors (includes IRF3 and NF-κB) are phosphorylated and transported to the nucleus to induce the production of IFN-β [14]. The IFN-β bind to respective cell-surface receptors on neighboring cells. Following the signal transduction, the phosphorylation of signal transducers and the activators of transcriptions 1 and 2 (STAT1/2) are promoted in the cytoplasm. STAT1/2 phosphorylation then combined with IRF9 to form the IFN-stimulated gene factor 3 (ISGF3). The ISGF3 complex translocated to the nucleus activates the expression of antiviral cytokines by binding to the IFN-stimulated response elements (ISREs) [15].

Alphaviruses have developed numerous mechanisms to evade the innate immune response of the host. The majority of alphavirus proteins exhibit various degrees of antagonism towards innate immunity through distinct mechanisms [11]. Nsp1 prevents DNA sensing and type I IFN response induction during infection [16]. Both nsP3 and nsP4 induce transcriptional shutdown in the host [17]. However, the intricate nature of the mechanism by which nsP2 antagonizes the immune response is multifaceted. Numerous reports have indicated that the IFN-I response can be counteracted through the inhibition of general transcription in host cells and the reduction of STAT1 phosphorylation in the JAK-STAT pathway [18–20]. Additionally, among the structural proteins, the capsid protein demonstrates a significant ability to impede RNA interference (RNAi) by separating double-stranded RNA and small interfering RNA [21, 22]. The E2 and E1 proteins hinder the activation of the IFN-β promoter, which is induced by the MDA5/RIG-I receptor signaling pathway [23].

At present, the mechanism of regulating the innate immune response by GETV infection and its role in pathogenesis is still unclear. In this study, we researched the sensitivity of GETV infection to IFN-β and the ability of antagonizing immune response, as well as confirmed the strongest inhibitor GETV nsP2 protein. Then, we respectively explored the mechanism of nsP2 suppressing IFN-β production and interfering IFN-β signaling pathway. Furthermore, the critical amino acids of nsP2 exerts its suppressive activity was determined, we also confirmed the importance of the key amino acids of nsP2 on the pathogenic of GETV.

## Materials and methods

### Cells, virus and reagents

Human embryonic kidney (HEK293A), Swine testicular (ST), and Baby hamster kidney (BHK-21) cell lines were cultured in Dulbecco’s modified Eagle medium (DMEM; Invitrogen) supplemented with 10% fetal bovine serum (FBS; Hyclone, USA) at 37 °C in an atmosphere with 5% CO_2_. GETV HuN1 strain (MF741771.1), GETV expressing green fluorescent protein (GETV-GFP), and Vesicular stomatitis virus expressing green fluorescent protein (VSV-GFP) were kept at our laboratory. Mouse anti-Flag monoclonal antibodies were obtained from Sigma (Sigma, USA). Rabbit anti-GAPDH mAb were purchased from Proteintech (Wuhan China). Rabbit anti-IRF3 mAb, rabbit anti-IRF3 phosphorylation mAb were purchased from Cell Signaling Technology (Boston, MA). Rabbit anti-STAT1 mAb, rabbit anti-STAT1 phosphorylation mAb, rabbit anti-JAK mAb, rabbit anti-TYK2 mAb, rabbit anti-STAT2 mAb, rabbit anti-STAT2 phosphorylation mAb, Rabbit anti-HA mAb, HRP-conjugated goat anti-mouse IgG, and HRP-conjugated goat anti-rabbit IgG were acquired from ABclonal (Wuhan, China). Dylight-conjugated 488 goat anti-mouse lgG and Dylight-conjugated 549 goat anti-rabbit lgG were purchased from Abbkine (Wuhan, China) GETV nsP2 mAb was kept at our laboratory. IFN-β and Poly (I:C) LMW (Low Molecular Weight) were purchased from InvivoGen (San Diego, CA).

### Plasmid constructs

The plasmid containing full length of GETV genome (pAC-GETV) were kept at our laboratory, and all GETV protein genes (nsP1, nsP2, nsP3, nsP4, E1, E2, E3,C, and 6 K) were amplified from the pAC-GETV plasmid, which contained the whole genome sequence of GETV. Viral genes were respectively cloned into the pCAGGS plasmid with 3×Flag tag at the N-terminal (pCAGGS-3Flag-nsP1, pCAGGS-3Flag-nsP2, pCAGGS-3Flag-nsP3, pCAGGS-3Flag-nsP4, pCAGGS-3Flag-E1, pCAGGS-3Flag-E2, pCAGGS-3Flag-E3, pCAGGS-3Flag-C, and pCAGGS-3Flag-6 K). NsP2 truncated genes (nsP2-M1, nsP2-M2, and nsP2-M3) were inserted into the pCAGGS-3Flag to constructed the expression plasmids contained different motifs of nsP2. The amino acids (K648, R649, and V650) of nsP2 gene were mutated to Alanine and also inserted into the pCAGGS-3Flag and constructed the expression plasmids (pCAGGS-3Flag-nsP2-M4, pCAGGS-3Flag-nsP2-M5, and pCAGGS-3Flag-nsP2-M6). Moreover, the nsP2, nsP2-M1, nsP2-M2, and nsP2-M3, nsP2-M4, nsP2-M5, and nsP2-M6 genes were respectively inserted into the pEGFP-C3. The primers used for the cloning were listed in Table 1. The sequence integrity of all constructs was confirmed by Sanger sequencing. The plasmids expressing innate immune genes and the pAC-GETV plasmid were kept in our laboratory.

**Table 1:** The sequences of the primers used for PCR

Primer	Sequence
nsP1 F	GCCACCATGAAGGTAACCGTGGACGTTGAGG
nsP1 R	TTAGGCTCCGGCTCTGAAGGTTAATTCC
nsP2 F	GCCACCATGGGGGTTGTGGAAACACCCAGG
nsP2 R	TTAACAGCCAGCAGTGTGCAATCCG
nsP3 F	GCCACCATGGCACCGTCATACAGGGTCCGCCGC
nsP3 R	TTACGCGCCAGCCCTGCCTAGTCAGG
nsP4 F	GCCACCATGTATATCTTTTCGTCTGACACTGG
nsP4 R	TTATTTAGGACCGCCGTACAGATG
C F	GCCACCATGAATTACATCCCAACTCAAACC
C R	TTACCATTCTTCTGTTCCTTCTGGGG
E3 F	GCCACCATGTCCGCCGCCTTGATGATGTGCG
E3 R	TTAGCGACGGTGGCGTGCACTATTG
E2 F	GCCACCATGAGTGTGACGGAACACTTCAATGTC
E2 R	TTAGGCATGCGCTCGTGGCGCGCAGC
6 K F	GCCACCATGGCGTCATTTGCAGAATCTATGGCG
6 K R	TTAAGATTTTACGACGGGAGTTCCC
E1 F	GCCACCATGTACGAACACACCGCAACGATCCCG
E1 R	TTAGCGGCGCATAGTCACACACGTCACC

###  Recovery of the nsP2 mutated GETV

The key amino acids mutated nsP2 genes were respectively replaced the nsP2 gene in the pAC-GETV. These mutated plasmids, namely pAC-GETV-nsP2-K648A, pAC-GETV-nsP2-R649A, and pAC-GETV-nsP2-V650A, were subjected to sequencing. BHK-21 cells were then seeded in 6-well plates and transfected with the respective mutated pAC-GETV plasmids. After 48 h of transfection, the cell culture supernatants from the BHK-21 cells were transferred to ST cells. The cytopathic effect (CPE) resulting from infection was monitored daily. The mutated viruses, designated as GETV-K648A, GETV-R649A, and GETV-V650A, were subsequently recovered. ST cells were infected with these mutated viruses at a multiplicity of infection (MOI) of 0.1. The culture supernatants were collected at 12, 24, 36, 48, and 60 h post-infection (hpi). The virus titer was assessed through cytopathic effect (CPE) observation and quantified as 50% tissue culture infective dose (TCID50) per milliliter (mL) using the Reed-Muench method.

### Reverse transcription-quantitative PCR (RT-qPCR)

According to the manufacturer’s instructions, the extraction of cells or the mice brain tissues total RNA were performed using Trizol reagent and treated with DNase I to remove contaminated DNAs. First-strand complementary DNA was synthesized from 1 µg of total RNA using a TransScript RT reagent kit. Uni-12, Uni-13, and oligo dT primers were used for reverse transcription of vRNA, cRNA, and mRNA, respectively. Oligo dT and random primers were used for detecting host genes. Generated cDNA was subjected to qPCR in a 20 µl reaction volume using gene-specific primers followed by RT-qPCR analysis on an Applied Biosystems 7500 PCR machine utilizing Superscript III and SYBR green. cDNA quantities were normalized to the reference genes (Pig-Rpl13a or Mouse-actin). To determine the copy numbers of virus RNA in the mice brain tissue, a pair of qPCR primers (E2 F and E2 R) was designed based on the conserved region of the GETV E2 gene. The GETV E2 gene was cloned into pCAGGS-MCS vector and used to draw the standard curve. The E2 gene copy number was calculated based on the standard curve, which was comprised of tenfold serial dilutions of the standard DNA. The primer sequences employed in this study can be found in Table 2.

**Table 2:** The sequences of the primers used for RT-qPCR

Primer	Sequence
Pig-Rpl13a F	CACGAGGTTGGCTGGAAGTA
Pig-Rpl13a R	GGCTGGTCCTTTGCCAGTTA
Pig-IFN-β F	GCTTGGATTCCTACAAAGAAGCA
Pig-IFN-β R	ATAGATGGTCAATGCGGCGTC
Pig-ISG15 F	CACCGTGTTCATGAATCTGC
Pig-ISG15R	CTTTATTTCCGGCCCTTGAT
Pig-ISG56 F	CCTCCTTGGGTTCGTCTACA
Pig-ISG56 R	GGCTGATATCTGGGTGCCTA
E2 F	AGCGACAAGACTATCAATTCGT
E2 R	TGCACTTTACCTTTGCGAGAC
Mouse-actin F	GAAATCGTGCGTGACATCAAAG
Mouse-actin R	TGTAGTTTCATGGATGCCACAG
Mouse-IFN-β F	CGTGGGAGATGTCCTCAACT
Mouse-IFN-β R	CTGAAGATCTCTGCTCGGACC
Mouse-TNF-α F	AGCCGATGGGTTGTACCTTG
Mouse-TNF-α R	ATAGCAAATCGGCTGACGGT
Mouse-IL-6 F	TTCCATCCAGTTGCCTTCTTGG
Mouse-IL-6 R	TTCTCATTTCCACGATTTCCCAG
Mouse-IFN-γ F	ATGAACGCTACACACTGCATC
Mouse-IFN-γ R	CCATCCTTTTGCCAGTTCCTC

### IFN reporter assay

The cells cultivated in 24-well plates were co-transfected with pRL-TK plasmid, which expressed Renilla luciferase (Rluc) and firefly luciferase (Fluc) reporter plasmids (pISRE-luc/pIFN-β-luc/pIRF3-luc/pNF-κB-luc), utilizing the Lipofectamine 3000 transfection reagent. Subsequently, the cells were either infected with viruses or transfected with plasmids. Following stimulation with Poly (I:C) or without stimulation, the cells were lysed with passive lysis buffer, and the luciferase activity in the lysates was determined with the Dual-Luciferase Reporter Assay System. The Renilla luciferase construct pRL-TK was simultaneously transfected as an internal control. The results are presented relative to those of untreated cells (mock-infected with viruses or transfected with a control plasmid).

### VSV-GFP interferon bioassay

ST cells were infected with GETV at a MOI of 0.1 or incubated with IFN-β at 10 U/ml for 0, 6, 12, and 18 h. Supernatants obtained from virus-infected cells were subjected to UV irradiation for 30 min to eliminate infectivity before conducting the bioassay. The ultraviolet radiation-inactivated supernatants were subsequently diluted in a 2-fold manner. Freshly grown ST cells were cultured in 96-well plates and exposed to 100 µl of each dilution for a duration of 12 h. Following this, the cells were infected with VSV-GFP at an MOI of 0.1 for 12 h, and the expression of GFP was assessed using inverted fluorescence microscopy.

### IFN sensitivity assay

In the context of IFN pretreatment, ST cells were cultivated in 24-well plates and subjected to varying concentrations of IFN-β for a duration of 12 h. Following this, the cells were rinsed and subsequently infected with GETV-GFP at a MOI of 0.1. Two hours after the viral absorption, the cells were washed again and incubated for an additional 20 h. In the case of IFN posttreatment, ST cells were infected with GETV-GFP at an MOI of 0.1. Two hours after viral absorption, the cells were exposed to diverse concentrations of IFN-β and allowed to incubate for an additional 20 h. The expression of GFP was assessed using inverted fluorescence microscopy.

### Flow cytometry analysis

ST cells cultured in 24-well plates were subjected to varying doses of IFN-β (1, 10, 100 U/ml) for a duration of 12 h. Subsequently, the cells were rinsed and exposed to GETV-GFP at a MOI of 0.1 for 20 h. The cells were then collected by treating them with 0.25% trypsin, followed by two washes with PBS at 1000 rpm for 5 min. The cells were finally suspended in PBS. The specific fluorescence emitted by GFP was assessed using the FL1 channel. A total of 10,000 events were recorded, and the resulting data was analyzed utilizing CytExpert Software 2.3.

### Western blotting

Cells were lysed in lysis buffer containing 1 mM phenylmethanesulfonyl fluoride (PMSF) and supplemented with phosphatase inhibitors for a duration of 30 min. Subsequently, the lysed cells were harvested. The resulting cell lysates were subjected to incubation at a temperature of 100 °C for a period of 5 min. The samples were then separated using SDS-PAGE and transferred onto polyvinylidene difluoride (PVDF) membranes for the purpose of immunoblotting. Following this, the membranes were incubated with a solution consisting of 5% nonfat dry milk in TBST (0.05% Tween-20) for a duration of 2 h. Subsequently, the membranes were incubated overnight at a temperature of 4 °C with the primary antibody. The membranes were treated with either HRP-conjugated goat anti-rabbit or HRP-conjugated goat anti-mouse secondary antibodies. Finally, the bands were visualized using NcmECL Ultra and imaged with the Tanon imaging system.

### Immunofluorescence assay

ST cells cultured on glass coverslips in 24-well plates were infected with either GETV or its mutation at a MOI of 0.1. After 24 h of infection, the cells were fixed in a solution of 4% formaldehyde in phosphate-buffered saline (PBS) for a duration of 10 minutes at room temperature. Subsequently, the cells were permeabilized using 0.1% Triton X-100 and blocked with a solution of 2% BSA in PBS for 30 minutes. Following this, the cells were sequentially stained with primary antibodies overnight at a temperature of 4°C. Secondary antibodies were then incubated with the cells for 1 hour at room temperature, and the nuclei were stained using 4’,6-diamidino-2-phenylindole (DAPI). Finally, the cells were observed under a fluorescent microscope.

### Animal experiments

The SPF ICR mice aged 3 to 4 days were categorized into 5 groups (*n* = 8 per group) and subjected to intracranial infection with 10^6^ TCID_50_ of GETV or GETV mutations. Mock mice were injected with DMEM. The weights, mortality, and health conditions of all mice were observed daily for a period of 15 dpi. Disease outcomes were assessed using a clinical score system, where no symptoms were recorded as 0, emaciation as 1, one hind limb paralysis as 2, both hind limb paralysis as 3, and death as 4.

### Statistical analysis

All statistical analyses were performed using GraphPad Prism software V8.0.1. Data are presented as the mean ± SD unless otherwise indicated. Differences between experimental and control groups were determined by the Student’s t test. For all tests, differences between groups were considered significant when the *P*-value was > 0.05 (ns), < 0.05 (*), < 0.01 (**) and < 0.001 (***). The Kaplan–Meier method was adopted for animal survival analysis to generate graphs, and the survival curves were analyzed with log-rank analysis.

## Results

### GETV Infection downregulate the production of IFN-β

**Fig. 1:**
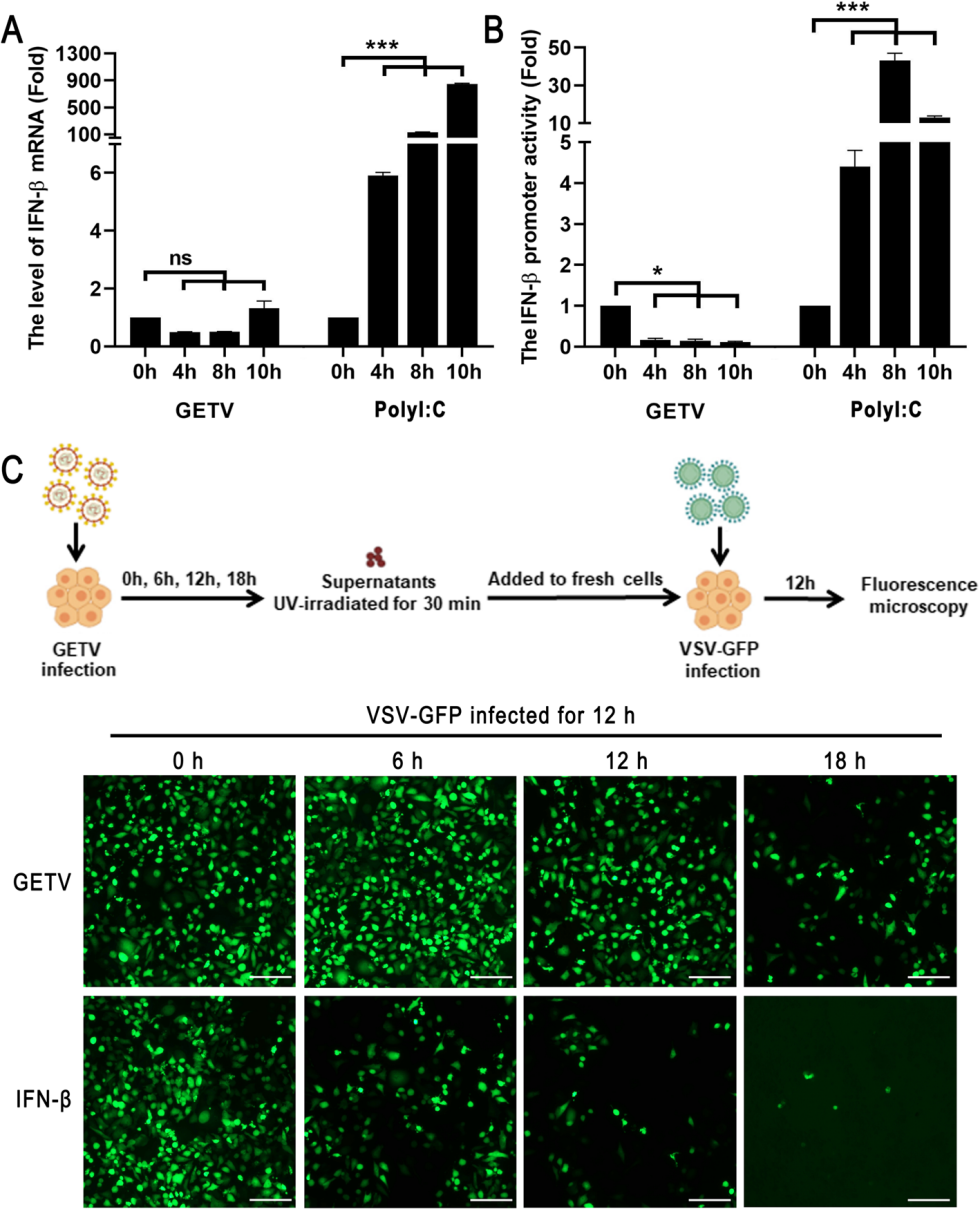
GETV infection downregulate the production of IFN-β. **A** ST cells were infected with GETV at a MOI of 0.1 or transfected with Poly (I:C) at 200 ng. The mRNA expression level of IFN-β was quantified at 0, 4, 8, and 10 h post-transfection using RT-qPCR (The house keeping gene was Rpl13a which was unaffected by IFNs). The presented data represents the outcome of three separate experiments. Statistical analysis revealed a highly significant difference (***P < 0.001), while “ns” denotes no significant difference. **B** ST cells were transfected with pIFN-β-Luc and pRL-TK for 24 h, followed by infection with GETV at a MOI = 0.1 or transfected with Poly (I:C) at 200 ng. The activity of the IFN-β promoter was assessed at 0, 4, 8, and 10 h post-infection. The presented data represents the outcome of three separate experiments. Statistical significance was denoted as *P < 0.05 and ***P < 0.001. **C** ST cells were infected with either GETV at a MOI of 0.1 or incubated with IFN-β at 10 U/ml for different time points (0, 6, 12, and 18 h). The supernatants obtained from the virus-infected cells were subjected to UV irradiation for 30 min to eliminate infectivity before conducting the bioassay. These supernatants were then diluted in a serial manner by 2-fold. Freshly grown ST cells were cultured in 96-well plates and incubated with 100 µl of each dilution for 12 h. Subsequently, the cells were infected with VSV-GFP (MOI = 1) for another 12 h, and the expression of GFP was examined using inverted fluorescence microscopy. The scale bar in the images represents a length of 100 μm. This analysis was replicated in two separate experiments, yielding consistent outcomes, and a representative fluorescence diagram was presented

To investigate the potential impact of GETV infection on IFN-β production, various aspects including IFN-β mRNA expression, IFN-β promoter activity, and IFN-β protein levels were examined in ST cells following GETV infection. The results depicted in Fig. 1A demonstrate a notable inhibition of IFN-β mRNA upregulation in GETV-infected cells, particularly at 4 hpi and 8 hpi. In contrast, stimulated with Poly (I:C) exhibited a significant induction of IFN-β mRNA expression. To further assess IFN-β promoter activity, ST cells were transfected with IFN-β-luciferase reporter plasmids and subsequently infected with GETV or transfected with Poly (I:C). Similarly, we observed a decrease in IFN-β luciferase activities in cells infected with GETV compared to the strong signal observed in cells transfected with Poly (I:C) (Fig. 1B). To further validate the inhibition of IFN-β production at the protein level caused by GETV infection, an IFN bioassay was conducted using an IFN-sensitive VSV-GFP. The cellular supernatants from IFN-β- triggered ST cells significantly inhibited the replication of VSV-GFP, whereas the presence of supernatants from cells expressing GETV partially restored the replication of VSV-GFP (Fig. 1C). The findings collectively indicate that infection with GETV downregulates the expression of IFN-β.

### GETV Infection inhibits the IFN-β-mediated signaling

**Fig. 2:**
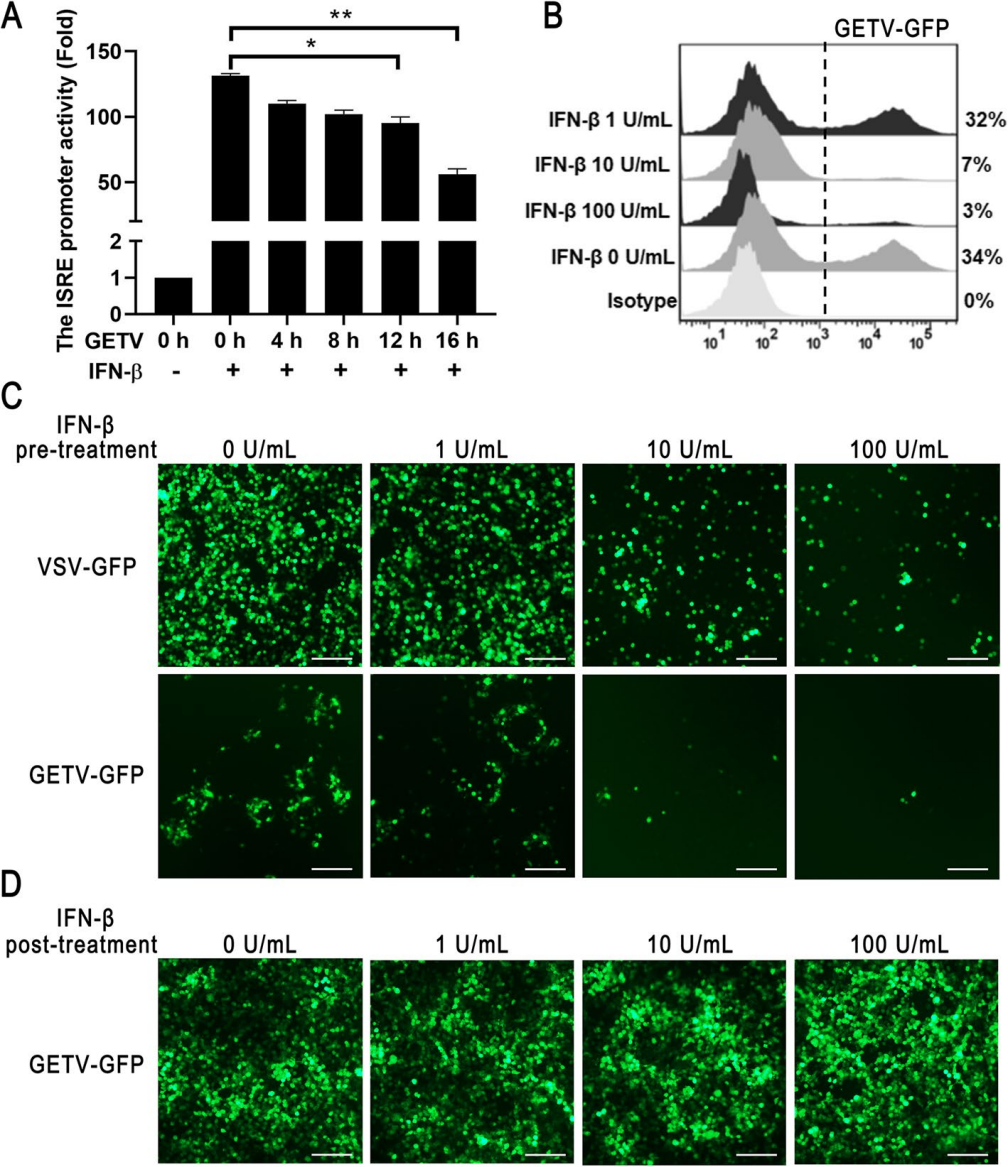
GETV infection inhibits the IFN-β signaling pathway.  **A** ST cells were transfected with pISRE-Luc and pRL-TK for 24 h. Subsequently, the cells were stimulated with IFN-β at a concentration of 100 U/ml. After a further 12 h, cell extracts were prepared to analyze the activity of the ISRE promoter. The data presented represents the outcome of three independent experiments. Statistical significance was determined with * *P*  < 0.05 and ** *P*  < 0.01. **B** and **C** ST cells were stimulated with varying concentrations of IFN-β (1, 10, and 100 U/ml) for 12 h. Following pre-treatment with IFN-β, the cells were then infected with GETV-GFP at a MOI of 0.1. After an additional 12 h, the expression of GFP was assessed using flow cytometry (FACS) and fluorescence microscopy. This analysis was replicated in two separate experiments, yielding comparable outcomes, and a representative fluorescence diagram was presented. **D** ST cells were infected with GETV-GFP (MOI = 0.1) for 12 h, followed by post-treatment with IFN-β at concentrations of 1, 10, and 100 U/ml. Subsequently, after an additional 12 h period, the expression of GFP was assessed through fluorescence microscopy. The scale bar, measuring 100 μm, was included. This analysis was conducted twice independently, producing consistent findings, and a representative fluorescence diagram was included

In order to investigate the impact of GETV infection on IFN-β-mediated induction of antiviral response, the ST cells were subjected to transfection with ISRE-luciferase reporter plasmids, followed by infection with GETV for varying durations (0, 4, 8, 12, and 16 h). The luciferase assay results indicated a gradual decrease in ISRE promoter activity with the progression of virus infection (Fig. 2A). Additionally, the extent to which GETV replication could be hindered in cells by pre-treatment with IFN-β was assessed. ST cells were exposed to IFN-β for 12 h prior to infection with GETV-GFP or VSV-GFP, and subsequent detection of green fluorescence was performed using flow cytometry and fluorescent microscope at 20 hpi. The infectivity of GETV was found to decrease in a concentration-dependent manner of IFN-β (Fig. 2B, C), similar to the replication of IFN-sensitive VSV. However, post-treatment with IFN-β did not have an impact on the production of GETV (Fig. 2D). Our findings show that GETV infection effectively suppresses the host’s antiviral response, including both viral sensing and IFN-β signaling pathways.

### Identification of GETV proteins inhibiting the host antiviral response

**Fig. 3:**
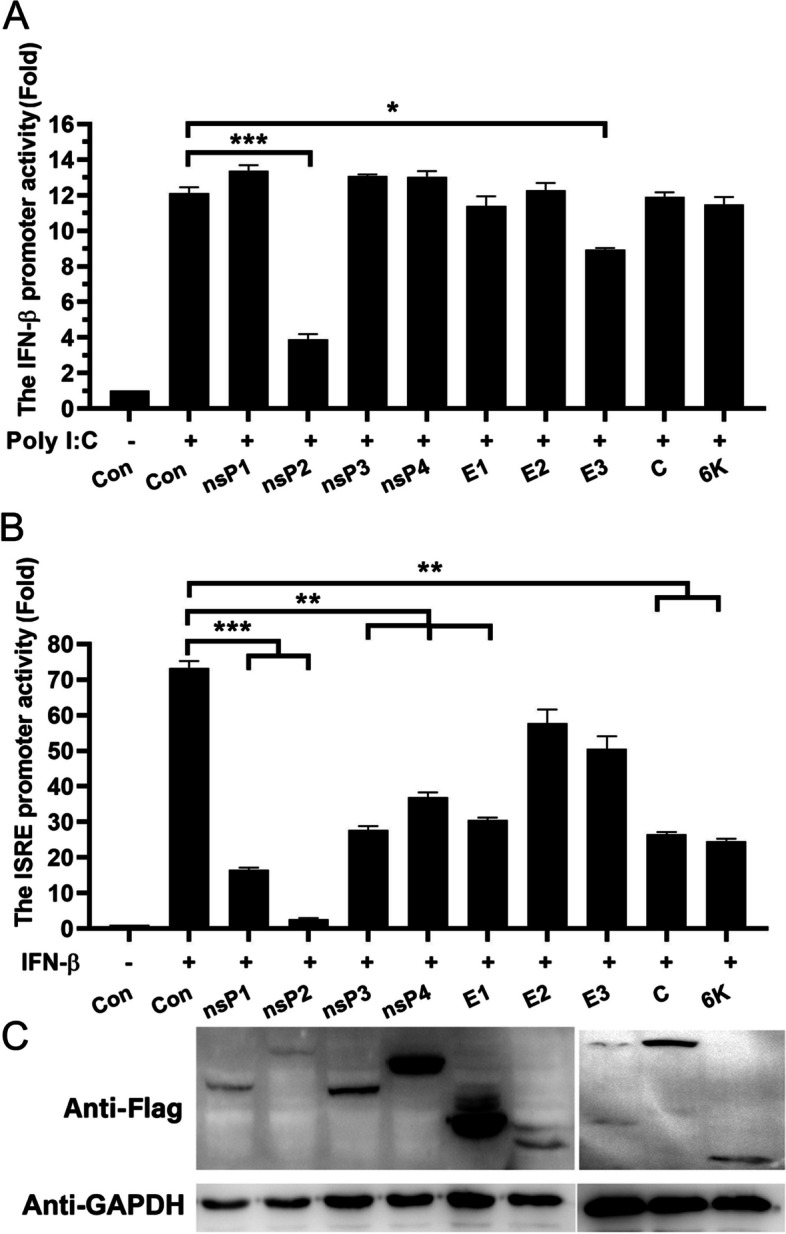
Identification of GETV proteins inhibiting the host antiviral response.  **A**, **B** HEK293A cells were transfected with recombinant plasmids encoding viral proteins, along with plasmids containing the luciferase reporter genes pIFN-β-Luc or pISRE-Luc, respectively. After 24 h of transfection, the cells were stimulated with Poly (I:C) (200 ng, 24 h) or IFN-β (100 U/ml, 12 h). Cell extracts were then prepared for analysis of luciferase reporter gene activity. The presented data represents the results of three independent experiments. Statistical significance was determined as follows: * *P*  < 0.05, ** *P*  < 0.01, and ***P < 0.001. **C** The Western blot was employed to detect the expression of the indicated GETV proteins

The research has demonstrated that the nsP2, E1, and E2 proteins of Chikungunya virus (CHIKV), which are classified within the genus alphavirus, possess significant antagonistic properties against the induction of IFN-β and IFN-β triggered antiviral response. We conducted an analysis to determine which viral protein in GETV is capable of inhibiting the host immune response. Recombinant plasmids were created to contain the genes for GETV E1, E2, E3, C, nsP1, nsP2, nsP3, nsP4, and 6 K. The expression of all viral proteins was confirmed using Western blot analysis (Fig. 3C). HEK293A cells were then co-transfected with plasmids expressing the viral proteins and IFN-β-luciferase reporter plasmids, the cells were treated with Poly (I:C). Our findings indicate that GETV-nsP2, in particular, significantly suppressed Poly (I:C)-mediated IFN-β promoter activity, while the other tested viral genes did not have the same effect (Fig. 3A). Subsequently, we employed the ISRE-luciferase reporter system to assess the efficacy of specific viral antagonists in inhibiting downstream events induced by IFN-β. Our findings consistently demonstrated that nsP2 exhibited the most significant inhibitory effect, resulting in a 70% reduction in IFN-β-induced ISRE promoter activity (Fig. 3B). These observations strongly indicate that GETV nsP2 functions as a primary viral antagonist, specifically targeting the Poly (I:C)-mediated induction of IFN-β and subsequent immune responses mediated by IFN-β.

###  GETV nsP2 suppresses IRF3 phosphorylation and nuclear transportation

**Fig. 4:**
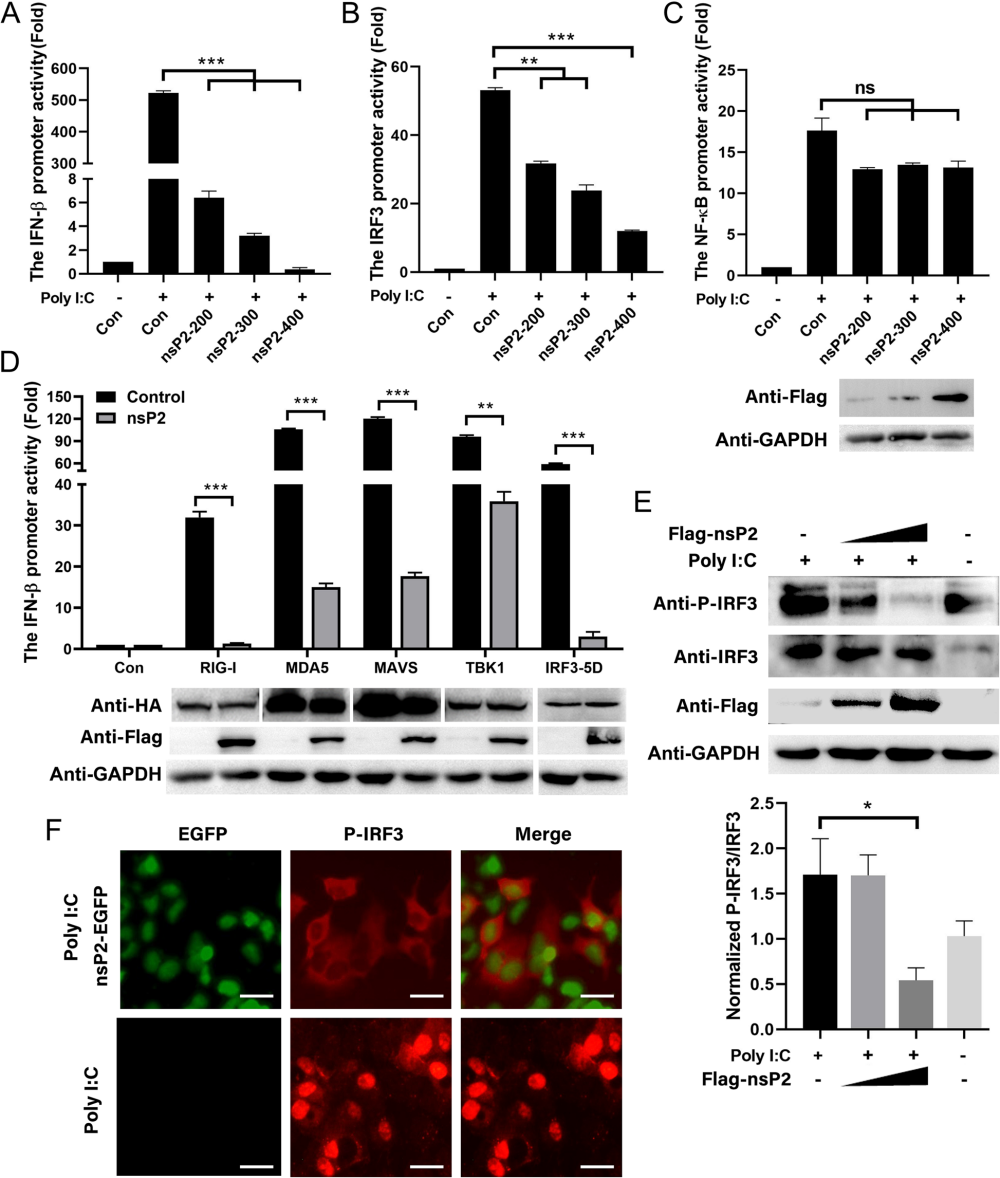
GETV nsP2 suppresses IRF3 phosphorylation and nuclear transportation.  **A**-**C** The different doses of pCAGGS-3Flag-nsP2 were transfected into HEK293A cells along with luciferase reporter gene plasmids (pIFN-β-Luc, pIRF3-Luc, pNF-κB-Luc). After 24 h of transfection, the cells were stimulated with Poly (I:C), and subsequently, the promoter activities were analyzed. The expression of Flag-nsP2 was assessed through Western blot. The presented data represents the findings from three independent experiments. Statistical significance was denoted as ** *P*  < 0.01, *** *P*  < 0.001, and ns indicated no significant difference. **D** The recombinant plasmids containing natural immune genes (RIG-I, MDA5, MAVS, TBK1 or IRF3-5D) were co-transfected with pCAGGS-3Flag-nsP2, pIFN-β-Luc, and pRL-TK into HEK293A cells. Following a 24 h incubation, the activation of the IFN-β promoter was analyzed. The expression of the natural immune proteins and nsP2 was assessed using Western blot. The presented data represents the findings of three independent experiments. Statistical significance was denoted as *** *P*  < 0.001. **E** HEK293A cells were transfected with varying concentrations of pCAGGS-3Flag-nsP2 for 24 h. Subsequently, the cells were subjected to stimulation with Poly (I:C) at a concentration of 200 ng for an additional 24 h. Equivalent quantities of cell lysates were subjected to Western blot analysis using antibodies specific to IRF3, P-IRF3, and Flag. The Western blot analysis was replicated in two separate experiments, yielding consistent outcomes. A representative blot was selected for presentation (upper), densitometry analysis of the ratio of P-IRF3/IRF3 on the immunoblots (lower). **F** Following transfection of pEGFP-nsP2 into HEK293A cells for 24 h, the cells were subsequently stimulated with Poly (I:C). Subsequently, the cells were fixed and subjected to analysis using the P-IRF3 antibody and the 549-conjugated goat anti-rabbit secondary antibody. The examination of IRF3’s nuclear translocation was carried out utilizing a fluorescence microscope. The scale bar, measuring 50 μm, was employed for reference. This experimental procedure was replicated twice independently, yielding consistent outcomes, and a representative fluorescence diagram was presented

In the context of IFN-β production, IRF3 and NF-κB are recognized as crucial transcriptional regulators. Research has indicated that the Sindbis virus (SINV) and Ross river virus (RRV) possess the ability to hinder the induction of type I IFN and the activation of IRF3 [24]. Consequently, the promoter activities of IRF3 and NF-κB were assessed using the luciferase reporter system after the expression of GETV nsP2. In comparison to Poly (I:C) stimulation, GETV nsP2 demonstrated a dose-dependent marginal inhibitory impact on the promoter activities of IFN-β (Fig. 4A) and IRF3 (Fig. 4B), while exhibiting no significant effect on NF-κB promoter activity (Fig. 4C).

In order to investigate the potential adaptor molecule for nsP2 in the IRF3 response, co-transfection of innate immune genes expression plasmids with pCAGGS-3Flag-nsP2 and luciferase reporter plasmids was conducted, followed by examination of IFN-β promoter activities and protein expression. The findings revealed that nsP2 inhibited the mediation of IFN-β promoter activities by these molecules, while having no significant impact on the overall quantity of innate immune genes (Fig. 4D). These results suggest that nsP2 hinders the activation of IRF3. IRF3, a crucial transcription factor, is promptly activated by various pathogen-associated molecular pattern receptors to initiate early transcription of IFN-I genes. Activation occurs through IRF3 phosphorylation, leading to protein dimerization and nuclear translocation. The phosphorylation of IRF3 in HEK293A cells was examined via Western blot analysis following pCAGGS-3Flag-nsP2 transfection and Poly (I:C) stimulation. Figure 4E demonstrates a significant reduction in IRF3 phosphorylation induced by Poly (I:C) with increasing nsP2 expression concentration. Furthermore, we generated the pEGFP-C3-nsP2 construct and transfected it into HEK293A cells. Subsequently, we employed indirect immunofluorescence to stain the phosphorylated IRF3. Upon Poly (I:C) stimulation, the overexpression of nsP2 resulted in a reduction of phosphorylated IRF3 in the cytoplasm. In contrast, the empty vector did not exhibit any inhibitory effect on the translocation of phosphorylated IRF3 into the nucleus (Fig. 4F). These findings suggest that GETV nsP2 acts as an antagonist to Poly (I:C)-mediated IFN-β production primarily by diminishing IRF3 activation.

### GETV nsP2 hinders the phosphorylation of STAT1 and its nuclear accumulation

**Fig. 5:**
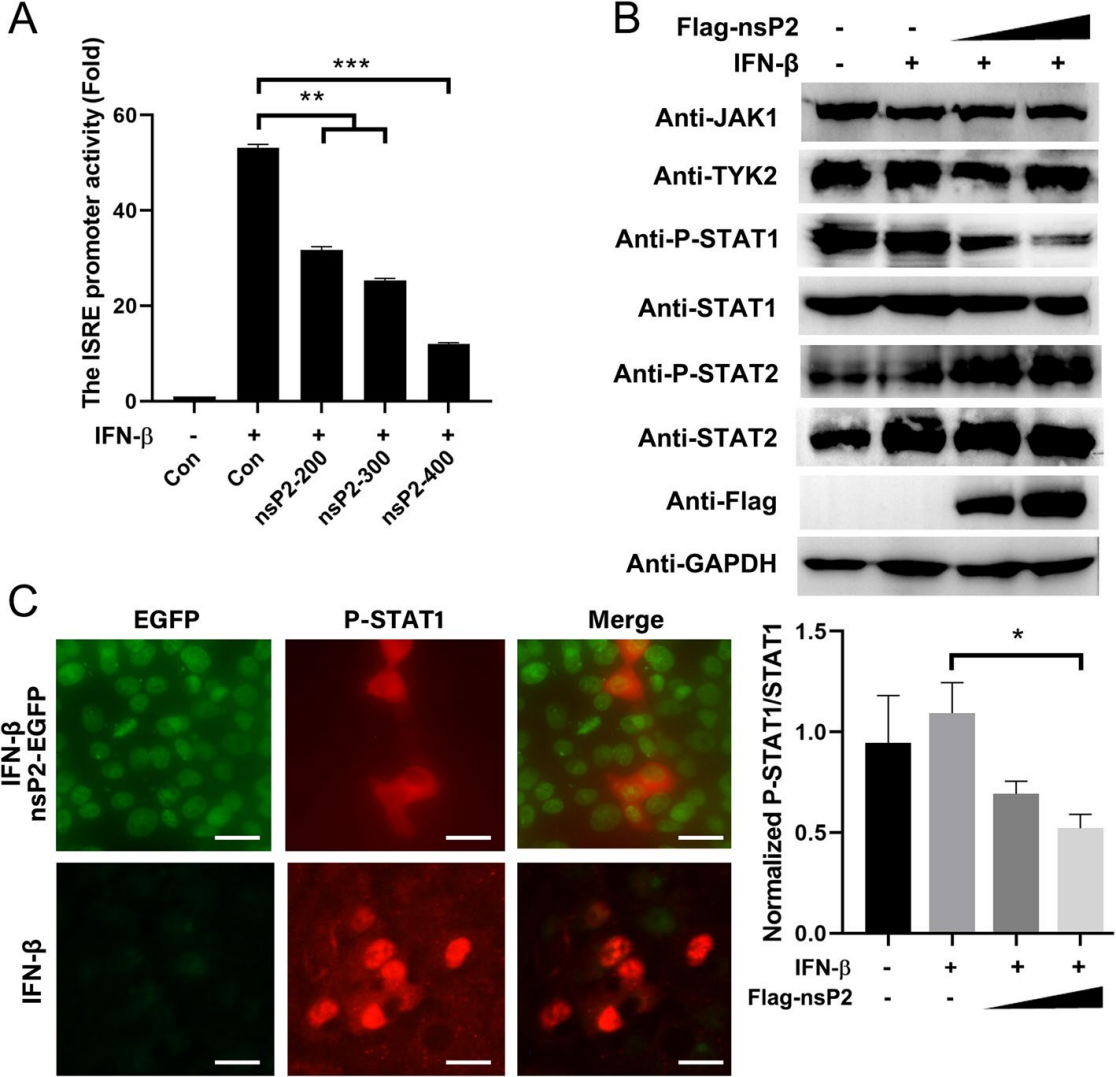
GETV nsP2 hinders the phosphorylation of STAT1 and its nuclear accumulation.  **A** The luciferase reporter gene plasmids (pISRE-Luc) were transfected into HEK293A cells with varying doses of pCAGGS-3Flag-nsP2. After 24 h of transfection, the cells were stimulated with IFN-β, and the analysis of ISRE promoter activities was conducted. The expression of Flag-nsP2 was detected through Western blot. The presented data represents the outcome of three independent experiments. Statistical significance was denoted as ** *P*  < 0.01 for highly significant, *** *P*  < 0.001 for extremely significant, and ns indicated no significant difference. **B** Following transfection of HEK293A cells with varying doses of pCAGGS-3Flag-nsP2, the cells were subsequently stimulated with IFN-β. Western blot analysis was conducted to detect the expression levels of JAK1, TYK2, STAT1, P-STAT1, STAT2, P-STAT2, and Flag-nsP2. This experimental procedure was replicated in two separate trials, yielding consistent outcomes, and a representative blot was presented (upper). Densitometry analysis of the ratio of P-STAT1/STAT1 on the immunoblots (lower). **C** The HEK293A cells were subjected to transfection with pEGFP-nsP2, followed by the evaluation of nuclear translocation of STAT1 using a fluorescence microscope. The scale bar, measuring 50 μm, was employed for reference. This experimental procedure was replicated twice independently, yielding consistent outcomes, and a representative fluorescence diagram was presented

In our study, we observed a dose-dependent decrease in ISRE promoter activities with increasing expression concentration of GETV nsP2 (Fig. 5A). To investigate the signaling pathway of IFN-β regulated by nsP2, we analyzed the levels of JAK1, TYK2, STAT1, STAT2, and their phosphorylated forms using Western blot. The relative abundance of phosphorylated protein to total protein was determined by densitometry. We found that phosphorylated STAT1 protein was significantly reduced in a dose-dependent manner after transfection with pCAGGS-3Flag-nsP2. Conversely, the expression of other proteins demonstrated an increase upon stimulation with IFN-β (Fig. 5B). We conducted an evaluation of the nuclear transportation of STAT1 in cells expressing 3Flag-nsP2 using a laser fluorescence microscope. Our observations revealed a significant decrease in nuclear STAT1 phosphorylation level in cells positive for nsP2, as compared to control cells expressing an empty vector (Fig. 5C). These findings provide evidence supporting the notion that the inhibition of STAT1 phosphorylation and nuclear transportation in the JAK/STAT signaling pathway by nsP2 may serve as a potential mechanism for GETV to evade the IFN-β response.

### The essential region of nsP2 suppressing the production and response of IFN-β

**Fig. 6:**
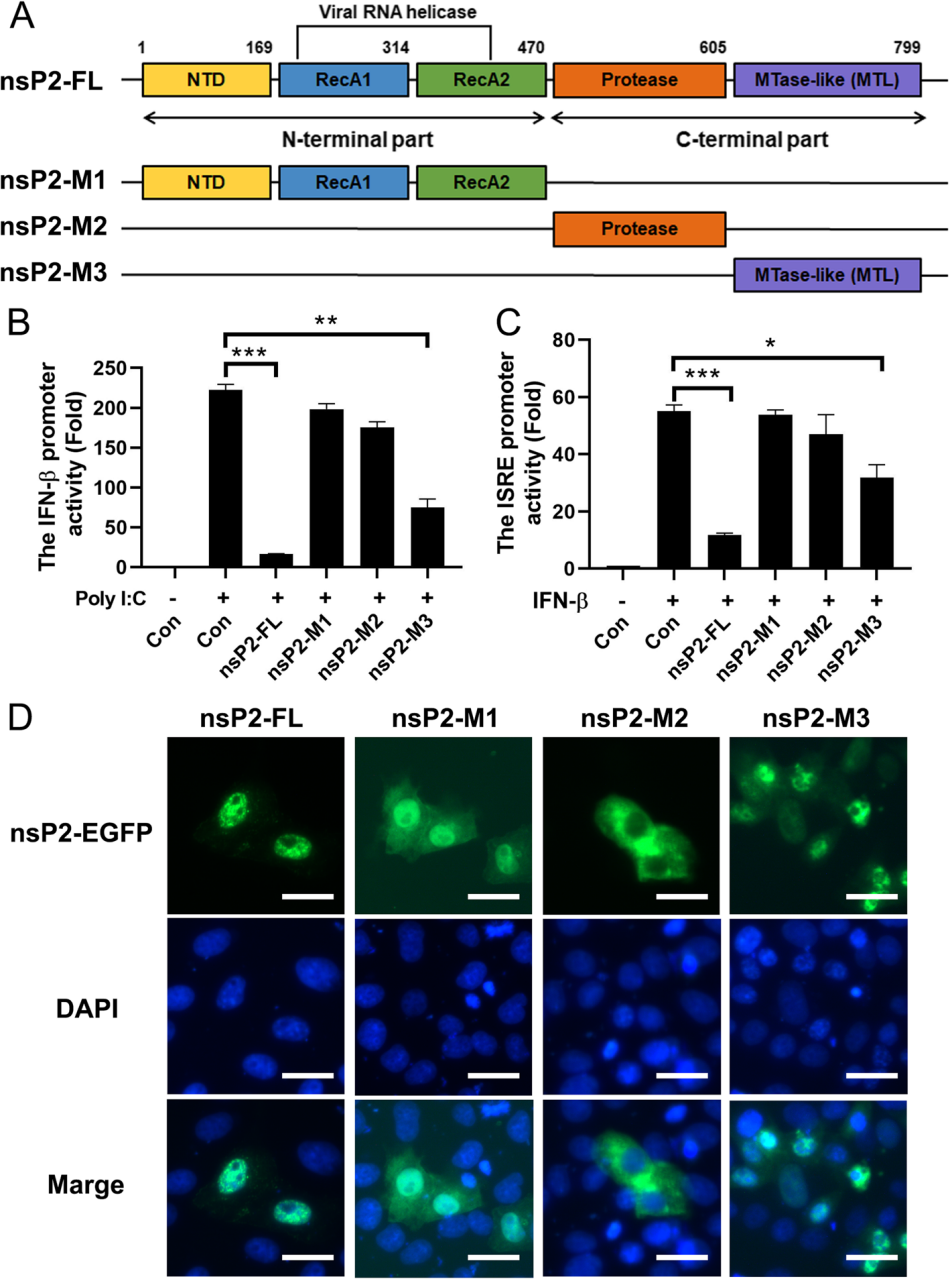
The C-terminus determines the nuclear localization of nsP2 and serves to inhibit the immune responses. **A** The schematic diagram presented in this study depicts the coding region of nsP2, highlighting its structural motifs. The N-terminal helicase domain of nsP2 comprises an N-terminal domain (NTD) and two RecA-like domains, which are potentially capable of NTP hydrolysis (RecA1 and RecA2). Additionally, the C-terminal region of nsP2 encompasses a papain-like protease (Protease) and a domain exhibiting similarity to the folding of methyltransferase (MTL). To investigate the functionality of nsP2, expression plasmids were constructed, incorporating various motifs (nsP2-M1, nsP2-M2, and nsP2-M3), along with either a Flag or EGFP tag positioned at the N-terminal of nsP2. **B** and **C** HEK293A cells were transfected with recombinant plasmids containing nsP2 and its truncations, along with plasmids containing the luciferase reporter genes pIFN-β-Luc or pISRE-Luc. After 24 h of transfection, the cells were stimulated with Poly (I:C) (200 ng, 24 h) or IFN-β (100 U/ml, 12 h). Cell extracts were then prepared for analysis of luciferase reporter genes. The data presented represents the results of three independent experiments. Statistical significance was determined as follows: * *P*  < 0.05, ** *P*  < 0.01, and *** *P*  < 0.001. **D** The pEGFP-nsP2-FL, pEGFP-nsP2-M1, pEGFP-nsP2-M2, and pEGFP-nsP2-M3 constructs were introduced into ST cells and allowed to incubate for a period of 24 h. Subsequently, the subcellular distribution of nsP2 and its truncated variants was assessed through the utilization of a fluorescence microscope. The scale bar provided in the images represents a length of 50 μm

The alphavirus nsP2 protein encompasses the viral helicase, protease, and a putative C-terminal methyltransferase domain, and it interacts with numerous host proteins [25]. Consequently, we hypothesized that the structure of GETV nsP2 could be elucidated by aligning its amino acid sequence (Fig. 6A). Despite the initial predictive analysis indicating the presence of these domains in GETV nsp2, their specific functions in terms of interferon antagonism and impact on the pathogenicity of GETV remain unknown. We divided nsP2 into three fragments, each containing distinct major motifs. The nsP2-M1 segment comprised an N-terminal domain (NTD) and two RecA-like domains, the nsP2-M2 segment contained a papain-like protease (Protease), and the nsP2-M3 segment resembled a methyltransferase-like folding (MTL). Our luciferase analysis revealed that the nsP2-M3 segment was capable of inhibiting the activation of IFN-β and ISRE promoters, whereas neither the nsP2-M1 nor nsP2-M2 segments exhibited this functionality (Fig. 6B, C). It has been previously documented that the C-terminal domain of alphavirus nsP2 exhibits a high degree of multifunctionality, being implicated in various cellular processes such as host cell shutoff and CPE, inhibition of JAK-STAT signaling, viral replication, and nuclear translocation [26]. GETV nsP2 was also localized in the nuclear (Figs. 4F and 5C), regardless of whether the two characteristics are intrinsically linked. We examined the subcellular localization of nsP2 truncations and found that only nsP2-M3 was exclusively localized to the nuclear (Fig. 6D), which retained nsP2 functionality. According to our research findings, the nuclear localization signal (NLS) of the GETV nsP2 protein is situated within the amino acid range of 645–650, although these results have not been published.

**Fig. 7:**
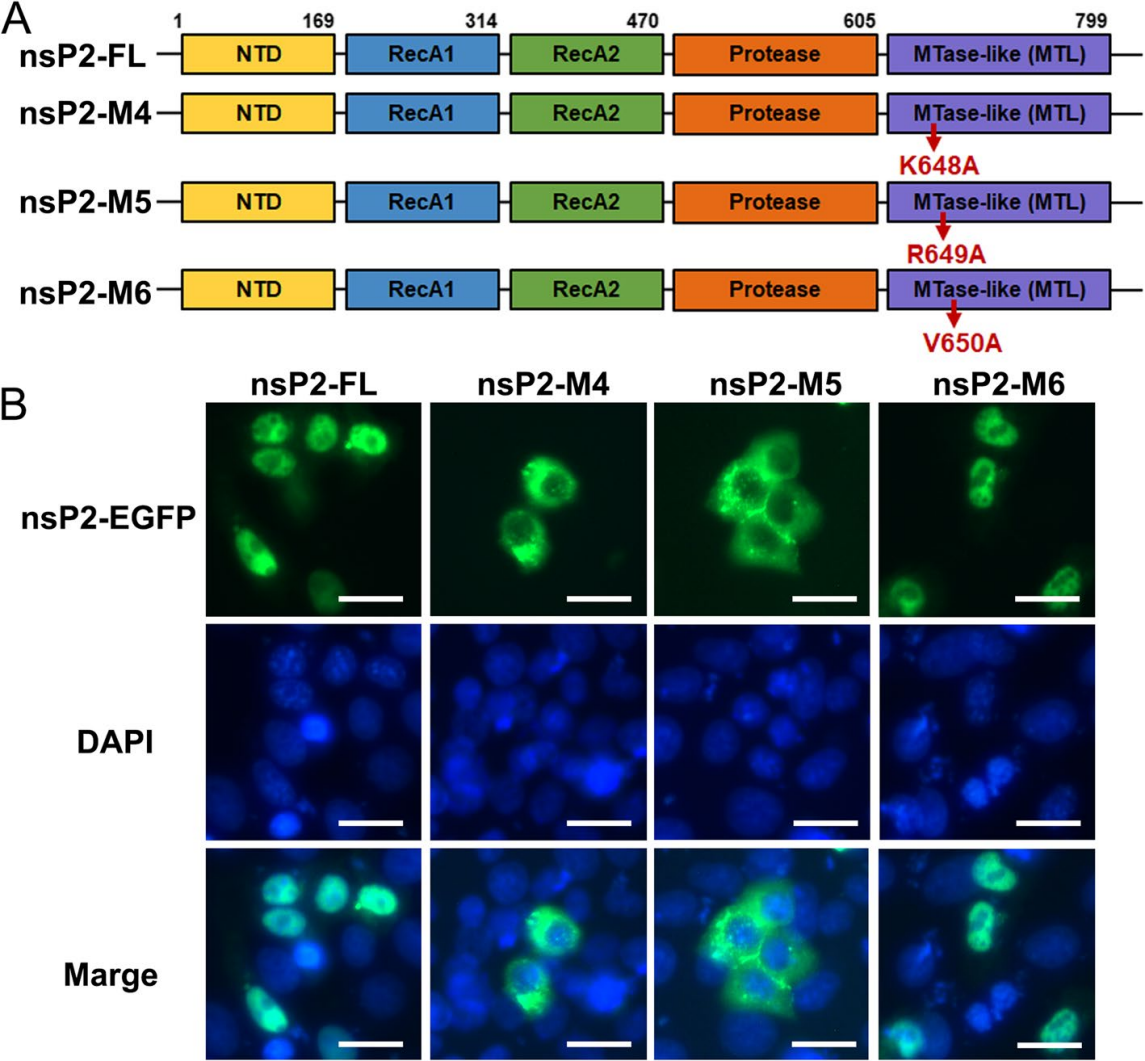
The amino acids K648 and R649 are associated with the nuclear localization of nsP2. **A** The amino acids K648, R649, and V6450 in nsP2, which are associated with nuclear localization, were subjected to arginine (A) mutations. Subsequently, the mutated nsP2 variants were cloned into pCAGGS-3Flag and pEGFP-C3 vectors. **B** The resulting constructs, namely pEGFP-nsP2-FL, pEGFP-nsP2-M4, pEGFP-nsP2-M5, and pEGFP-nsP2-M6, were transfected into ST cells for a duration of 24 h. Following this, the subcellular localization of nsP2 and its mutated forms was assessed using a fluorescence microscope. The scale bar in the images represents a length of 50 μm

**Fig. 8:**
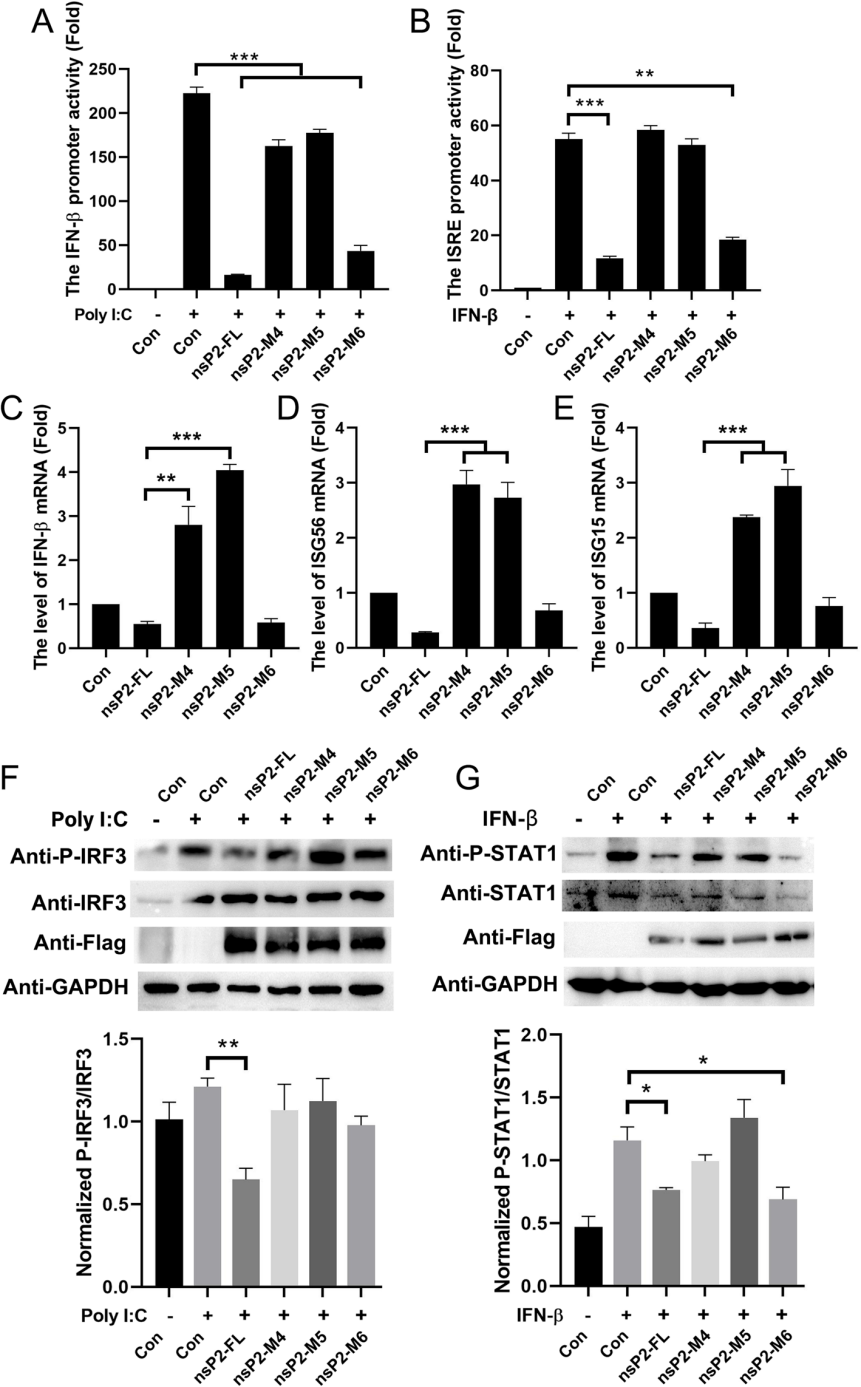
The immune responses were inhibited by the amino acids K648 and R649 of nsP2. **A** and **B** Recombinant plasmids containing nsP2 and its mutations were transfected into HEK293A cells, along with luciferase reporter plasmids (IFN-β-Luc and ISRE-Luc). After 24 h of transfection, the cells were stimulated with Poly (I:C) or IFN-β, and subsequently, the promoter activities were analyzed. The presented data represents the outcome of three independent experiments. Statistical significance was denoted as ** *P*  < 0.01 and *** *P*  < 0.001. **C**-**E** HEK293A cells were transfected with recombinant plasmids containing nsP2 and its mutations. After a 24 h, the mRNA levels of IFN-β, ISG15, and ISG56 were measured using RT-qPCR. The data presented represents the results obtained from three independent experiments. Statistical analysis revealed significant differences with ** *P*  < 0.01 and *** *P*  < 0.001. **F** and **G** HEK293A cells were transfected with pCAGGS-3Flag-nsP2-FL, pCAGGS-3Flag-nsP2-M4, pCAGGS-3Flag-nsP2-M5, and pCAGGS-3Flag-nsP2-M6 plasmids, followed by stimulation with Poly (I:C) or IFN-β. The expression levels of IRF3, P-IRF3, STAT1, and P-STAT1 were determined using Western blot analysis (upper). Densitometry analysis of the ratio of P-IRF3/IRF3 and P-STAT1/STAT1 on the immunoblots (lower)

Subsequently, in accordance with our studies on the NLS of GETV nsP2, and the key nuclear localization residues observed in other alphavirus nsP2 [19, 26], we introduced mutations at positions K648, R649, and V650, replacing them with alanine residues in the GETV nsP2-M3 (Fig. 7A). The pEGFP-nsP2-M4, pEGFP-nsP2-M5, and pEGFP-nsP2-M6 plasmids were constructed. Our observations indicate that the mutation of amino acids K648 and R649 to alanine resulted in a complete loss of nsP2 nuclear localization, as shown in Fig. 7B. Additionally, we conducted an analysis to investigate the role of nuclear localized amino acids of nsP2 in the inhibition of immune responses. It was found that nsP2-M6 (V650A) exhibited strong suppression of Poly (I:C)-mediated IFN-β and IFN-β-mediated ISRE promoter activities, similar to nsP2-FL. However, nsP2-M4 (K648A) and nsP2-M5 (R649A) did not demonstrate the same inhibitory ability (Fig. 8A and B). The RT-qPCR analysis of IFN-β, ISG15, and ISG56 provided further evidence to support the notion that the K648A and R649A mutations induce the expression of antiviral factors (Fig. 8C-E). Additionally, in order to investigate the impact of the nsP2 NLS on the activation of IRF3 and STAT1, it was observed that nsP2-FL and nsP2-M6 led to a reduction in the phosphorylation of IRF3 and STAT1, particularly STAT1 phosphorylation, whereas nsP2-M4 and nsP2-M5 no longer exhibited inhibitory activity (Fig. 8F and G). Collectively, these findings highlight the critical role of K648 and R649 in facilitating nsP2 nuclear translocation and its ability to suppress the host immune response.

### The rescue of GETV mutants and examination of their biological characteristics

**Fig. 9:**
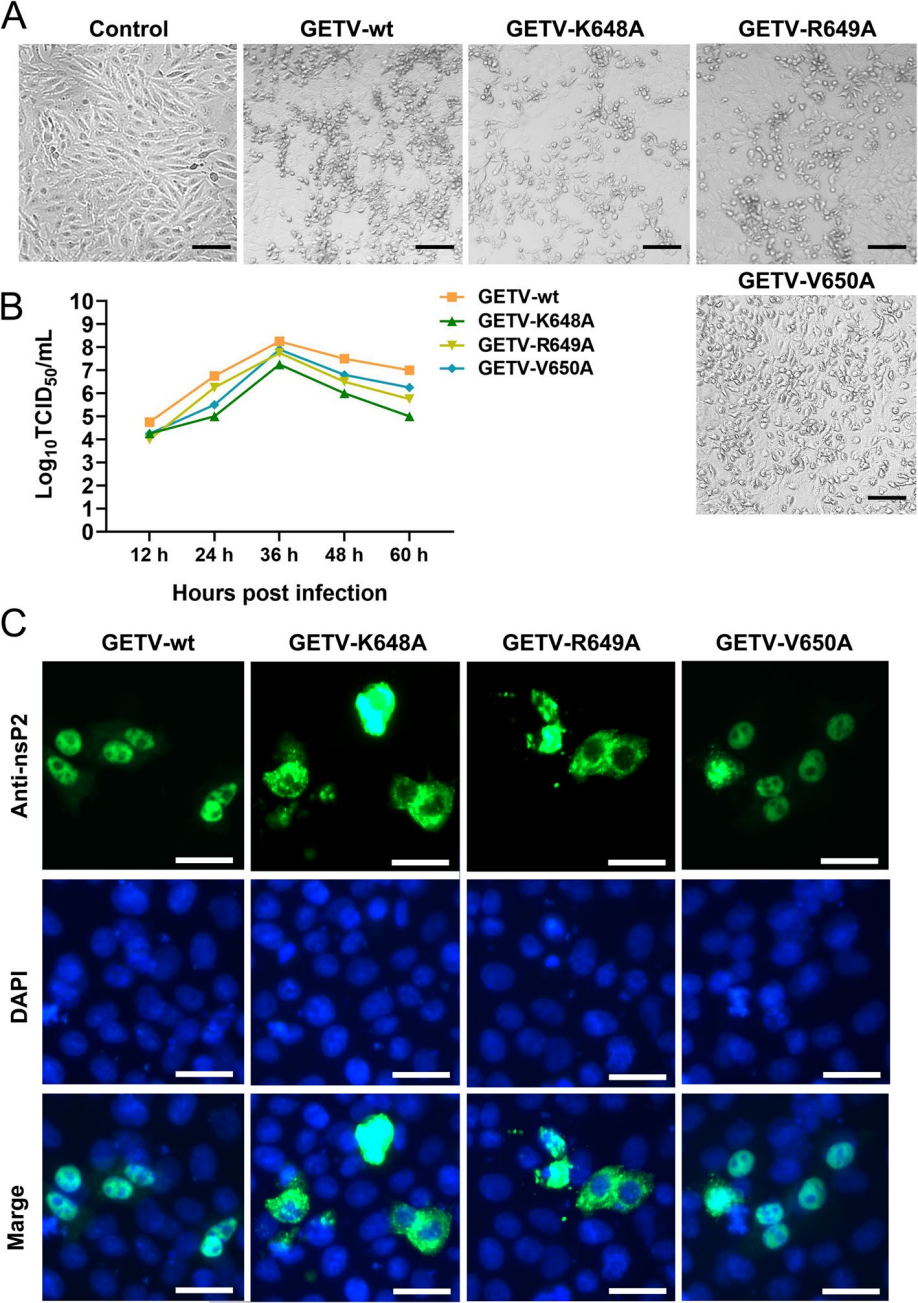
The rescue of GETV mutants and examination of their biological characteristics. **A** The plasmids pAC-GETV-nsP2-K648A, pAC-GETV-nsP2-R649A, and pAC-GETV-nsP2-V650A were synthesized and subsequently introduced into BHK-21 cells via transfection. After 48 h, the cell culture supernatants were transferred to ST cells. The cytopathic effect (CPE) was monitored after an additional 24 h. The scale bar in the image represents a length of 50 μm. **B** The ST cells were infected with either GETV or the mutated viruses (GETV-K648A, GETV-R649A, and GETV-V650A) at a multiplicity of infection (MOI) of 0.1. The culture supernatants were collected at 12, 24, 36, 48, and 60 h, respectively. The viral titer was determined by observing the cytopathic effect and quantified as the 50% tissue culture infective dose (TCID 50 ) mL −1 . The presented data is indicative of the outcomes obtained from three distinct experiments. **C** The ST cells were infected with GETV or mutated viruses at a MOI of 0.1 for a 12 h. The subcellular localization of nsP2 was assessed by employing a fluorescence microscope and employing anti-nsP2 antibody staining. The scale bar, measuring 50 μm, was utilized for reference

Due to the significant role played by K648 and R649 in the function of nsP2, recombinants of GETV were generated wherein alanine replaced K648, R649, and V650 of nsP2. The cytopathic effects (CPE) of these GETV mutants were observed using a microscope (Fig. 9A). To assess the growth properties of the GETV mutants, one-step growth experiments were conducted. The GETV mutants exhibited a comparable growth cycle, with final titers at 60 h approximately 10–20 times lower than that of the wild-type GETV. Notably, the K648A mutant displayed even lower titers (Fig. 9B). In addition, we conducted immunofluorescence analysis employing a monoclonal antibody specific to the nsP2 protein of GETV to investigate the nuclear transport of nsP2 following infection with GETV mutants. As depicted in Fig. 9C, the nsP2 protein in cells infected with GETV-K648A and GETV-R649A exhibited cytoplasmic resistance.

### The crucial role of nsP2 K648 and R649 in the inhibition of immune responses by GETV

**Fig. 10:**
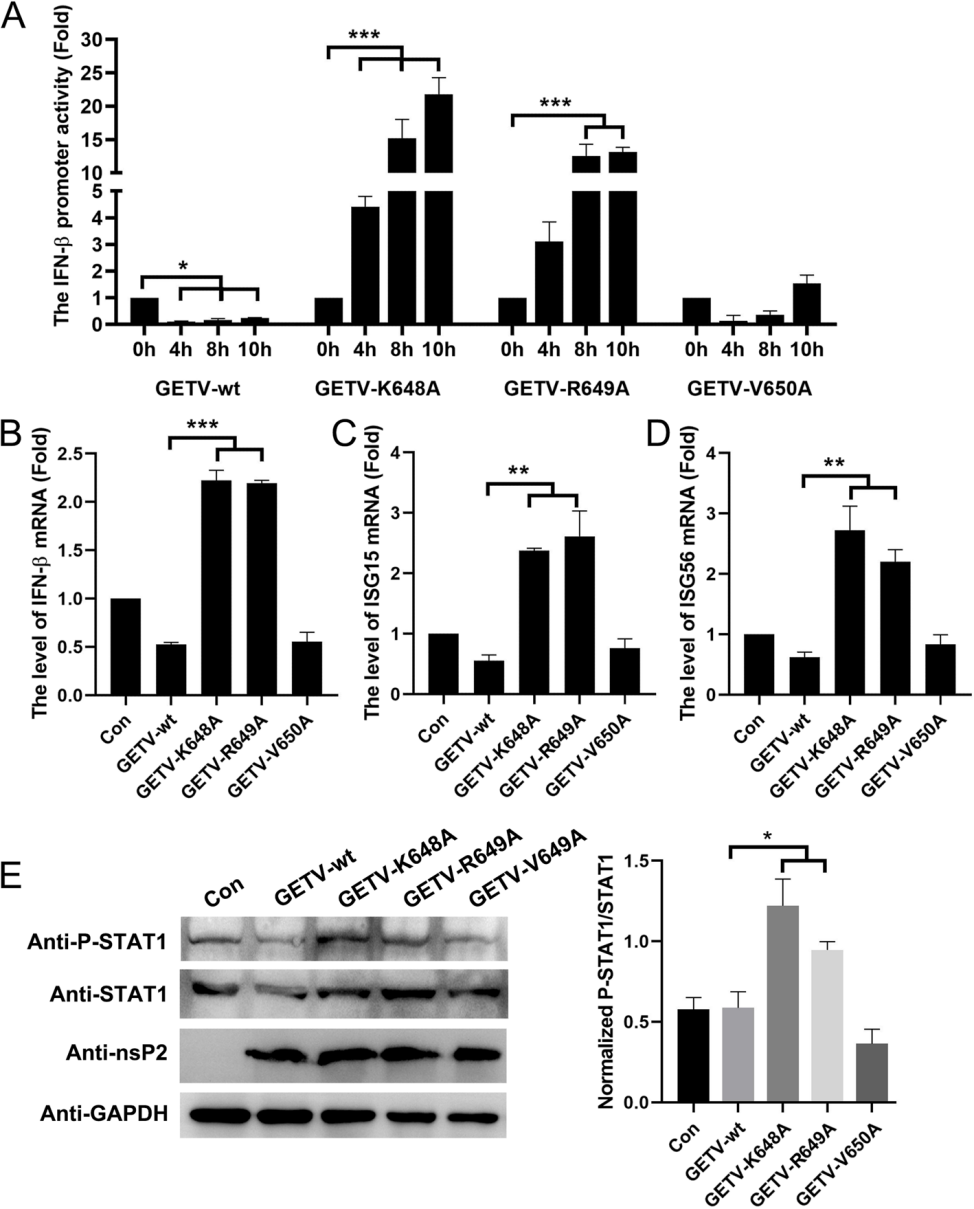
The crucial role of nsP2 K648 and R649 in the inhibition of immune responses by GETV.  **A** ST cells were transfected with pIFN-β-Luc and pRL-TK, followed by infection with GETV or mutated viruses (GETV-K648A, GETV-R649A, and GETV-V650A) at a MOI of 0.1. The activity of the IFN-β promoter was assessed at 0, 4, 8, and 10 h post-infection. The presented data represents the findings of three independent experiments. Statistical significance was determined as follows: * *P*  < 0.05, ** *P*  < 0.01, *** *P*  < 0.001. **B**-**D** ST cells were infected with GETV or mutated viruses (GETV-K648A, GETV-R649A, and GETV-V650A) at an MOI of 0.1, and the mRNA levels of IFN-β, ISG15, and ISG56 were measured by RT-qPCR at 12 h post-infection. The presented data represents the outcomes obtained from three separate experiments, with statistical significance indicated as ** *P*  < 0.01 and *** *P*  < 0.001. **E** ST cells were subjected to either GETV infection or mutation for 12 h, followed by the detection of STAT1, P-STAT1, and nsP2 expression through Western blot analysis (left). Densitometry analysis of the ratio of P-STAT1/STAT1 on the immunoblots (right)

In order to assess the significance of the nuclear localized amino acid of nsP2 in GETV’s evasion of immune responses, ST cells were infected with both wild-type GETV and viral mutants. The IFN-β promoter activities were then measured at various time intervals. After conducting the experiment four times, it was observed that both GETV-wt and GETV-V650A were still able to suppress the IFN-β promoter activities, consistent with our previous findings (Fig. 1A). However, GETV-K648A and GETV-R649A exhibited a partial loss of their inhibitory capability (Fig. 10A). The RT-qPCR analysis of IFN-β and ISGs production also provided confirmation that the V650 amino acid mutation in GETV-nsP2 has negative effects on the virus’s ability to interfere with the activation of the cellular antiviral response. Additionally, the K648A and R649A mutations in GETV-nsP2 resulted in approximately a three-fold reduction at 12 hpi (Fig. 10B-D). Given that the nsP2 NLS significantly reduces STAT1 phosphorylation in the JAK/STAT signaling (Fig. 8G), we further examined the level of STAT1 phosphorylation in cells infected with the aforementioned mutations at 12 hpi. The phosphorylation level of STAT1 was observed to be lower in cells infected with GETV-wt and GETV-V650A, as compared to cells infected with GETV-K648A and GETV-R649A (Fig. 10E). These findings indicate that the NLS (K648 and R649) located in the C-terminal region of nsP2 may play a role in the antagonistic effect of GETV on immune responses.

#### The pathogenicity of GETV necessitates the presence of nsP2 K648 and R649

**Fig. 11:**
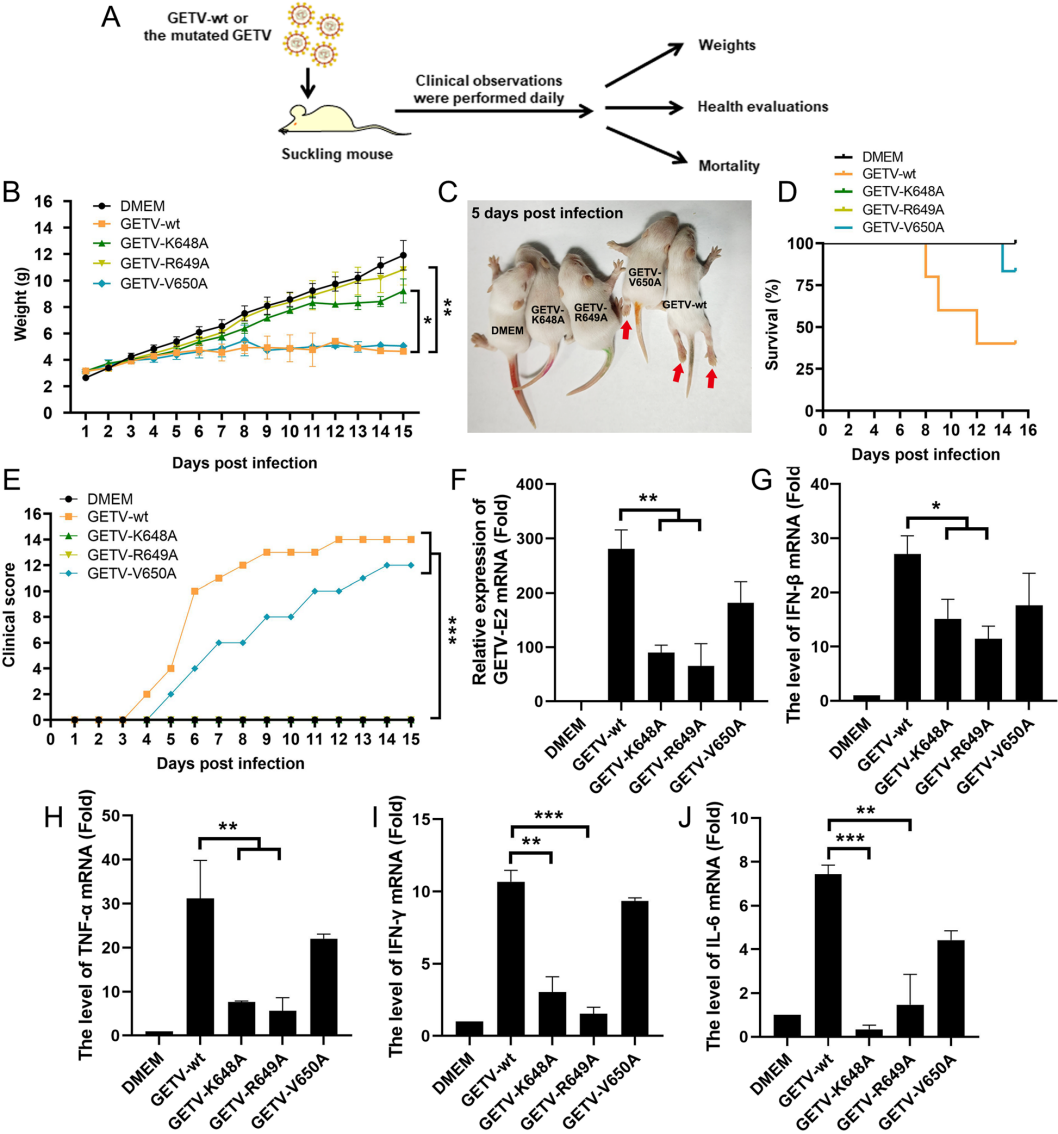
The pathogenicity of GETV necessitates the presence of nsP2 K648 and R649.  **A** A schematic depicting the infection of mice and subsequent health evaluations is presented. Mice were intracranially infected with 10 6 TCID 50 of either GETV or GETV mutations, while mock mice received injections of DMEM. **B**-**E** The weights, mortality rates, and health evaluations of all mice were monitored daily for a period of 15 days post-infection. Disease outcomes were recorded using a clinical score system, with scores ranging from 0 (no symptoms) to 4 (death), including intermediate scores for emaciation and hind limb paralysis. Statistical significance was determined using the symbols * *P*  < 0.05, ** *P*  < 0.01, and *** *P*  < 0.001. **F**-**J** The viral load, IFN-dependent and inflammatory cytokines (IFN-β, IFN-γ, TNF-α, and IL-6) mRNA levels in the brain tissue of mice at 7 days post infection were detected by RT-qPCR. The presented data represents the outcomes obtained from three separate experiments, with statistical significance indicated as ** *P*  < 0.01 and *** *P*  < 0.001

In order to investigate the potential impact of nuclear localized amino acids of nsP2 on the pathogenicity of GETV in vivo, a group of 3-4-day-old ICR suckling mice were infected with GETV and subjected to mutations (Fig. 11A). Through daily monitoring, it was observed that the group infected with GETV-wt and GETV-V650A experienced progressive weight loss (Fig. 11B), hind limb paralysis (Fig. 11C), and mortality (Fig. 11D). Conversely, the mice infected with GETV-K648A and GETV-R649A were able to maintain a healthy state similar to that of normal mice, exhibiting a low disease score (Fig. 11E). Furthermore, the levels of viral load, IFN-dependent and inflammatory cytokines (IFN-β, IFN-γ, TNF-α, and IL-6) mRNA were assessed in the brain tissue of mice at 7 days post infection, also revealing a diminished pathogenicity of GETV-K648A and GETV-R649A (Fig. 11F-J). Altogether, the aforementioned data indicate that the nuclear localization of the amino acid in nsP2 may significantly impede the pathogenicity of GETV in murine subjects.

## Discussion

Over the past few decades, numerous studies have been conducted to investigate the molecular mechanisms underlying the pathogenesis of various alphaviruses. These studies have focused on elucidating processes such as adsorption and cell entry, innate immune evasion, and the impact on cell apoptosis [11, 27, 28]. The accumulating body of evidence suggests that innate immunity plays a crucial role in the response to alphavirus infection [11]. Simultaneously, alphaviruses have developed multiple strategies to circumvent the host’s antiviral innate immune response. However, to date, there have been no definitive reports on GETV regulating the host innate immune response, as well as a dearth of identified proteins acting as antagonism factors for GETV infection. This study presents novel findings that elucidate the impact of GETV infection on the induction and response of IFN-β, a pivotal viral protein involved in regulation, as well as the underlying mechanism and key protein domain. Consequently, we have gained valuable insights into the mechanism by which GETV evades the host’s innate immune system.

It has been reported that several viral proteins within the alphavirus genus possess the capability to antagonize interferon. The nsP2, E1, and E2 proteins of CHIKV demonstrated a significant ability to counteract the activation of the IFN-β production. When nsP2, E1, E2, and MDA5/RIG-I were co-expressed, they effectively inhibited over 80% of the MDA5/RIG-I-mediated IFN-β promoter activity in the presence of viral proteins [11]. The palmitoylated TF proved to be advantageous in augmenting its capacity to counteract the host interferon response during Sindbis virus (SINV) infection [29]. Various viral proteins of GETV had also been observed to impede the activities of the IFN-β and ISRE promoters. Notably, our comprehensive investigation has unequivocally established that GETV nsP2 exerts the most potent antagonistic influence (Fig. 3). In the research of SINV and Semliki Forest virus (SFV), mutations in nsP2 displayed severe defects in counteracting the IFN response and resulted in high IFN production [30]. It was also observed that the mutation of GETV nsP2 resulted in an augmentation of IFN-β expression and a reduction in IFN-β signal pathway. These findings provide confirmation of the crucial involvement of GETV nsP2 in the modulation of IFN production and response.

The alphavirus nsP2 is a multifunctional protein and plays a crucial role in establishing persistent replication in mammalian cells. It is intricately involved in the shutoff of host macromolecular synthesis and also exerts influence on inducing cell death and inhibiting the release of IFN-I through its involvement in general host shutoff. Alphavirus nsP2 has the ability to induce the degradation of the RNA polymerase II subunit RPB1, leading to a global shutdown of host cell transcription and subsequently reducing ISG expression [26]. The occurrence of CHIKV nsP2 mutants with shutoff defects led to a decrease in the cytopathic effect (CPE) commonly observed with nsP2 expression, along with other alphaviruses such as SINV and SFV [19, 26, 31]. In GETV nsP2 expression cells, the Renilla luciferase activity expressed from a constitutive promoter decreased approximately 1.5 fold compared to that in mock-transfected cells at 24 h. For longer transfection time (more than 36 h), GETV nsP2 could cause mild cytopathic effects, indicating that GETV nsP2 resulted in some host shutoff. However, the firefly luciferase expression of the responsive element IFN-β, IRF3, or ISRE were markedly decreased (more than 10 fold) with the nsP2 transfection dose increases. Therefore, the relative firefly luciferase expression of IFN-β, IRF3, or ISRE (normalized to Renilla luciferase expression) were significantly inhibited in cells transfected with nsP2. Furthermore, certain individuals have conducted investigations into the crucial amino acids responsible for determining the functionality of nsP2 shutoff. These studies have revealed that mutations in CHIKV-nsP2 (P718S) and SINV-nsP2 (P726S), while still capable of rendering alphavirus replicons noncytopathic, exhibit a notable decrease in their ability to inhibit JAK-STAT signaling [32]. The relative firefly luciferase expression of NF-κB (Fig. 4) and the expression of host IFN-I signaling pathway proteins, including JAK1, TYK2, and STAT2, remained unaffected in cells expressing GETV nsP2 (Fig. 5). This finding suggests that the suppression of host antiviral response by GETV nsP2 is consistent with previous reports, that inhibition of IFN signaling is not attributable to host shutoff mechanisms [20, 32].

The currently available data regarding induction of IFN-I by different alphaviruses are fragmented and contradictory. It had been observed that MDA5 was the major PRR for SINV and SFV, and its deletion reduced IFN production from infected cells [33, 34]. The findings of the CHIKV studies indicated that RIG-I seemed to have a greater capacity than MDA5 for inducing IFN-I in primary fibroblasts infected CHIKV [35]. Furthermore, it is worth noting that IRF3 also plays a crucial role in the production of IFN-I following CHIKV infection [36]. There was an absence of detectable serum IFN-I and a significant increase in the titers of CHIKV, in IRF3^−/−^ mice [37]. The nsP2 protein of CHIKV demonstrated the capability to impede the activation of IRF3 and the activation of the IFN-β promoter activities mediated by IRF3-5D [23]. In our study, it was observed that GETV nsP2 acted as a potent antagonist against various signaling molecules (MDA5, RIG-I, MAVS, TBK1, and IRF3-5D) induced IFN-β production. Moreover, GETV nsP2 demonstrated the ability to hinder IRF3 phosphorylation and nucleation, as depicted in Fig. 4. It is tempting to postulate that IRF3 is the target of GETV nsP2 suppressing the IFN-β induction pathway, which was consistent with the behavior of CHIKV nsP2. We further revealed that the effect of GETV nsP2 on IRF3 activity is not significantly correlated with its nuclear localization, and the specific inhibition of IRF3 phosphorylation is not caused by inhibition of transcriptional translation of nsP2.

The IFN signaling pathway was effectively modulated by alphavirus nsP2 through the inhibition of phosphorylated STAT1 levels in the nucleus [38]. The nsP2 proteins of CHIKV and SINV also hindered the JAK-STAT signaling pathway by excluding STAT2 from the nucleus [39]. In cells expressing GETV nsP2, the phosphorylation of STAT1 and its accumulation in the nucleus were reduced. This indicted that phosphorylated STAT1 is responsible for the inhibition of IFN-β response by the nsP2 protein of GETV. Notably, the inhibitory capacity of GETV nsP2 was contingent upon the presence of nuclear localization amino acids situated within the C-terminal MTase-like domain. The confirmation of the involvement of this domain in the antagonism of alphavirus nsP2 against IFN-I has been established. By combining multiple alphaviruses to inhibit IFN-β response, the findings of the study suggest that the mechanism of GETV nsP2 in relation to the JAK-STAT signaling pathway and its associated protein binding sites exhibits a relatively conserved nature.

Experimentally, the injection of GETV into suckling mice via the intracranial route resulted in hind limb paralysis and mortality. Notably, there was a discernible disparity in the pathogenicity of GETV when its mutation (GETV-K648A and GETV-R649A) was introduced in the mouse model. The mutant strains exhibited reduced pathogenicity, as evidenced by the absence of mortality among infected mice (Fig. 11). Previous studies have attributed variations in virulence among alphaviruses to alterations in their structural proteins [40]. It is worth noting that the nuclear localization of nsP2 is a prerequisite for the cytotoxic properties observed in Old World alphaviruses [41]. In GETV-K648A or GETV-R649A, a single amino acid substitution in the nsP2 nuclear localization residues has been observed to lead to a reduction in the toxicity of GETV. K648A and R649A are a consequence of the exclusion of nsP2 from the nucleus, resulting in enhanced accumulation of STAT1 within the cell nucleus and weakened ability to antagonize IFN-β. This observation indicates that the replication and virulence of GETV are closely related to the innate immune response of the host. These findings suggest that the mechanism underlying the attenuation of GETV virulence in vivo is closely linked to the evasion of the innate immune response, as previously proposed for other alphaviruses.

## Conclusions

Our research offers a comprehensive understanding of the mechanisms employed by GETV nsP2 to evade the innate immune response, specifically by targeting the production and signaling pathway of IFN-β. Our findings demonstrate that GETV nsP2 employs effective strategies to evade the immune response by targeting two crucial protein molecules, IRF3 and STAT1, through the inhibition of their phosphorylation and nuclear translocation. Furthermore, we have identified that the specific NLS amino acid in the C-terminal region of nsP2, plays a pivotal role in determining the inhibition of the innate immune response and the pathogenicity of the GETV.

### Supplementary Information

**Additional file 1.**

## Data Availability

All datasets generated and/or analysed during the current study are presented in the article, the accompanying Source Data or Supplementary Information files, or are available from the corresponding author upon reasonable request.
